# Improving Outcomes in Catheter-Directed Thrombolysis for the Management of Acute Budd-Chiari Syndrome: A Case Report

**DOI:** 10.7759/cureus.35976

**Published:** 2023-03-10

**Authors:** Abhilasha Rana, Sriram Jaganathan, Brijesh Ray, Venkatram Krishnan

**Affiliations:** 1 Department of Radiology, Vardhman Mahavir Medical College and Safdarjung Hospital, Delhi, IND; 2 Department of Radiology, Aster Medcity, Kochi, IND

**Keywords:** acute budd-chiari syndrome, acute liver failure (alf), angioplasty and stenting, venous angioplasty, catheter-directed thrombolysis, hepatic vein thrombosis

## Abstract

Traditionally catheter-directed thrombolysis is performed for recanalization of hepatic vein thrombosis in acute Budd-Chiari syndrome. Successful recanalization of the hepatic veins requires a continuous infusion of the thrombolytic agent for an adequate duration due to increased resistance to blood flow in the setting of luminal thrombosis. Here, we describe a case of acute Budd-Chiari syndrome in a young female in whom prolonged catheter-directed thrombolysis of the right hepatic vein was performed for a duration of 84 hours using alteplase as the thrombolytic agent. This was followed by angioplasty and stent placement. We observed that prolonged catheter-directed thrombolysis was associated with a progressive reduction in clot burden with improved luminal patency of the hepatic vein and improved outcome of subsequent angioplasty and stenting. There was a rapid improvement in liver function tests after the procedure and liver enzymes returned to baseline within a week. A follow-up ultrasound scan showed normal blood flow and a patent lumen of the right hepatic vein. In the absence of complications, prolonged catheter-directed thrombolysis in acute Budd-Chiari syndrome can achieve adequate recanalization of the hepatic veins and improved long-term clinical outcomes. This may obviate the need for other invasive procedures like TIPS (transjugular intrahepatic portosystemic shunt)/DIPS (direct intrahepatic portosystemic shunt) and liver transplantation.

## Introduction

Budd-Chiari syndrome (BCS) includes a group of disorders characterized by hepatic venous outflow obstruction leading to an increase in hepatic sinusoidal pressure and ultimately portal hypertension. The anatomical site of obstruction can be at the hepatic veins or inferior vena cava (IVC) [1]. BCS is a rare condition with an incidence of one in 100,000 [2]. Clinically it can present as acute thrombosis or chronic disease. BCS rarely presents with acute hepatic failure when there is occlusion of all three major hepatic veins and insufficient time for the development of collaterals. Also, BCS is one of the rare causes of acute hepatic failure [3]. Acute disease is usually due to underlying hypercoagulable states such as thrombotic disorders, pregnancy, and the use of oral contraceptive pills [1,4]. Traditionally, treatment options included portosystemic shunting for stable patients and liver transplants for fulminant hepatic failure and chronic disease. In recent times, catheter-directed thrombolysis has become a viable option [4]. This is the most physiological option to treat acute BCS as it recanalizes the thrombosed hepatic veins and restores hepatic venous flow [4]. This should be done promptly before the development of chronic thrombus and loss of luminal caliber [5]. Here, we have presented a case of acute hepatic failure that was referred to our institution for liver transplantation. Pre-transplant cross-sectional imaging evaluation revealed acute thrombosis of all three hepatic veins. Prompt intervention with prolonged hepatic vein thrombolysis, angioplasty, and stenting was performed. This restored liver function and averted the need for liver transplantation, thereby avoiding expensive transplant surgery and various transplant-related complications. This case is unique due to the rarity of the presentation of BCS as an acute hepatic failure and prompt management of the same with a minimally invasive technique which resulted in good clinical outcome. We have also detailed our institutional protocol-based approach for the management of BCS.

## Case presentation

**Figure 1:**
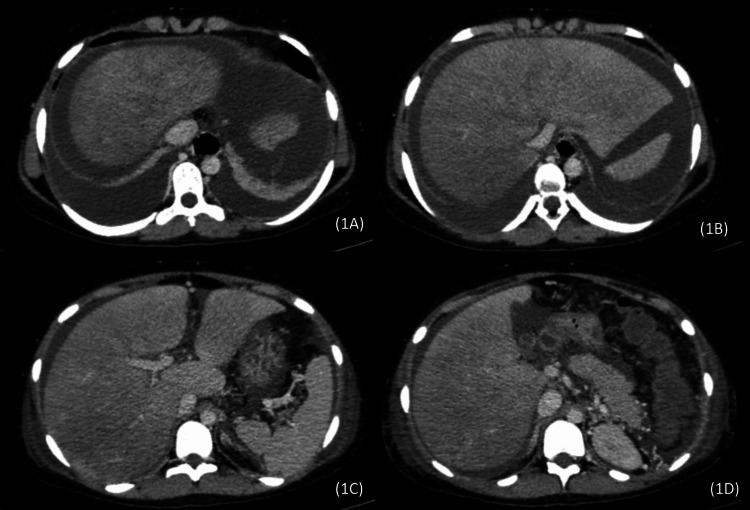
Contrast-enhanced axial abdominal CT images (1A, 1B, 1C, and 1D) show non-visualization of right, middle, and left hepatic veins, heterogeneous enhancement of liver parenchyma predominantly affecting the right lobe, normal caliber and luminal opacification of IVC and portal vein, gross ascites and bilateral pleural effusion. IVC, inferior vena cava; CT, computed tomography.

A 17-year-old non-pregnant female presented with progressively severe abdominal distension, abdominal pain, fatigue, jaundice, fever, and confusion for five days. There was no history of hematemesis, oral contraceptive use, or any known thrombotic disorder. Examination revealed tenderness in the right upper quadrant, shifting dullness, and icterus. Laboratory investigations showed elevated liver function tests (aspartate transaminase [AST] 424 U/L, alanine transaminase [ALT] 874 U/L, total bilirubin 6 mg/dL, international normalized ratio [INR] 5) and low hemoglobin level (9.8 mg/dL). Ultrasound of the abdomen showed hepatosplenomegaly with gross ascites. Viral markers were negative; autoimmune markers, serum ceruloplasmin, and iron and ferritin levels, including cardiac evaluation, were normal. Based on the clinical signs and symptoms, presence of encephalopathy, and acutely deteriorating liver function, she was diagnosed with acute hepatic failure of unknown etiology and a liver transplant was offered as the only viable treatment option. A repeat ultrasound abdomen with Doppler showed absent flow in the hepatic veins which raised the suspicion of hepatic vein occlusion. She was then referred to the radiology department for an abdominal computed tomography (CT) scan for evaluation of the hepatic vasculature and a pre-transplant work-up. CT scan revealed heterogeneous enhancement of the hepatic parenchyma with predominant involvement of the right hepatic lobe. There was non-visualization of the right, middle, and left hepatic veins, concerning hepatic venous thrombosis. IVC and portal vein were normal in caliber and contrast opacification. CT also showed ascites and bilateral pleural effusion (Figure 1A-1D).

**Figure 2:**
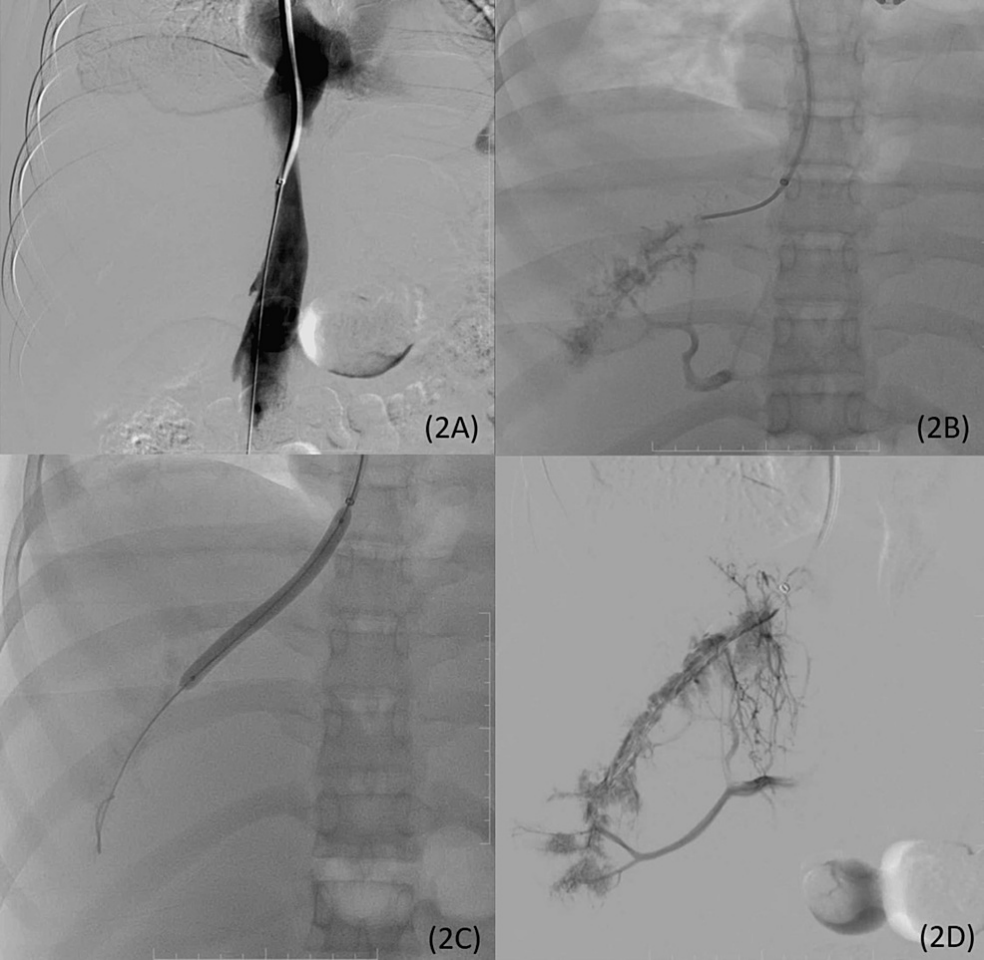
IVC venogram showing normal caliber and luminal opacification of intrahepatic and suprahepatic IVC without any focal narrowing (2A); right hepatic venogram showing diffuse irregular filling defects due to intraluminal thrombus with filling of surrounding small intrahepatic veins and contrast reflux in the portal venous branches (2B); balloon angioplasty with inflated balloon in the right hepatic vein to re-establish some luminal patency before administration of alteplase (2C); post-angioplasty venogram with thrombolysis catheter show slightly better luminal opacification of right hepatic vein (2D). IVC, inferior vena cava.

A diagnosis of acute BCS resulting in acute hepatic failure was made. Interventional radiology management for the recanalization of the thrombosed hepatic veins was planned after a multidisciplinary team meeting. It was decided to recanalize the right hepatic vein because of its good caliber, straight course, and CT changes showing predominant involvement of the right hepatic lobe. Venous access to the IVC was obtained through a transjugular approach. IVC venogram showed normal intrahepatic and suprahepatic IVC. A subsequent venogram of the right hepatic vein showed diffuse irregular filling defects consistent with an acute thrombus. Associated contrast reflux into the portal venous branches was visualized. Balloon angioplasty was initially performed to allow sufficient space to pass the thrombolysis catheter and enable better intra-luminal administration of the thrombolytic agent. Post-angioplasty venogram showed slightly better luminal opacification of the right hepatic vein (Figure 2A-2D).

The standard multipurpose catheter was modified on the table into a multi-holed infusion catheter using a 23G needle. The thrombolysis catheter was then left in situ and alteplase was infused. The initial infusion was performed at a rate of 0.5 mg/hour for 48 hours. The subsequent infusion was continued at a rate of 0.25 mg/hour. Fibrinogen levels were monitored every 6 hours and used to guide thrombolytic infusion. Alteplase was discontinued at a fibrinogen level of less than 150 mg/dL. A subtherapeutic dose of intravenous heparin was started simultaneously and continued for a duration of six days to maintain activated partial thromboplastin time (aPTT) less than 1.5 times the institutional reference range.

**Figure 3:**
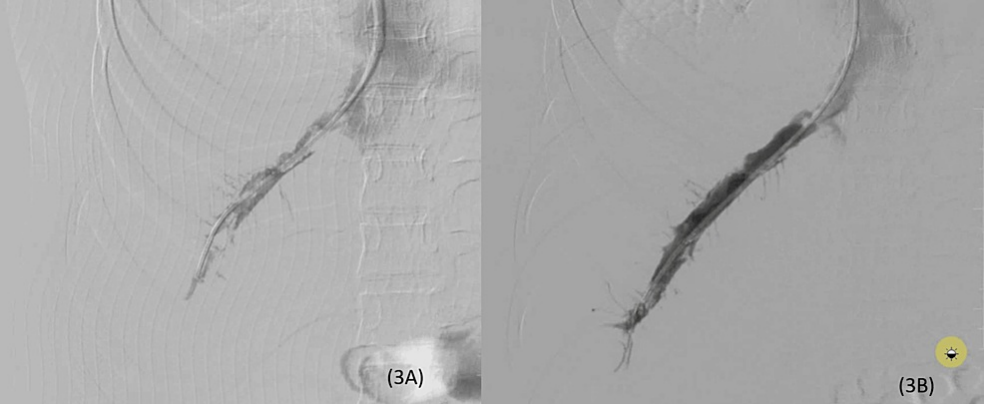
Check venogram at 36 hours (3A) and 48 hours (3B) post-thrombolysis show improved luminal patency and non-opacification of the small intrahepatic veins.

Check venograms after 36 and 48 hours showed a progressive reduction in clot burden with improved luminal patency (Figure 3A, 3B).

**Figure 4:**
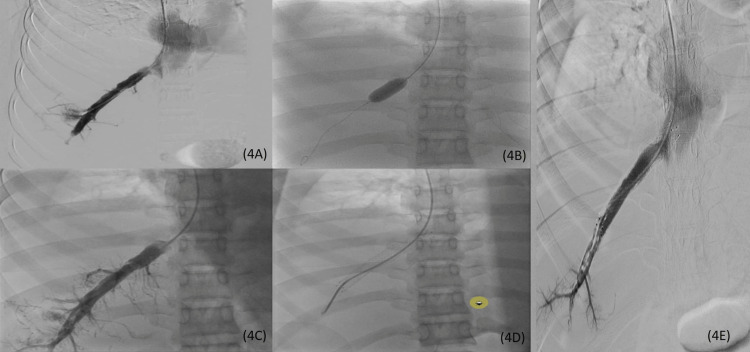
Venogram performed 84 hours post-thrombolysis showing patent lumen of the right hepatic vein and non-opacification of the small intra-hepatic veins with minimal luminal narrowing at the ostium of right hepatic vein (4A); balloon angioplasty image shows inflated balloon in the ostium of right hepatic vein (4B); post-angioplasty venogram shows normal caliber at the ostium with patent lumen (4C); post-angioplasty stent placement image shows catheter in right hepatic vein with stent in situ (4D); post-stenting venogram shows patent lumen and normal opacification of right hepatic vein (4E).

The infusion was continued for a total of 84 hours. The final venogram post-thrombolysis showed near-complete resolution of the thrombus with the patent lumen of the right hepatic vein and non-opacification of the small intra-hepatic veins. A total of 31 mg of alteplase was used for thrombolysis. Persistent short segment narrowing of about 40% at the ostium of the right hepatic vein was treated with balloon angioplasty followed by stent placement (Figure 4A-4E).

**Figure 5:**
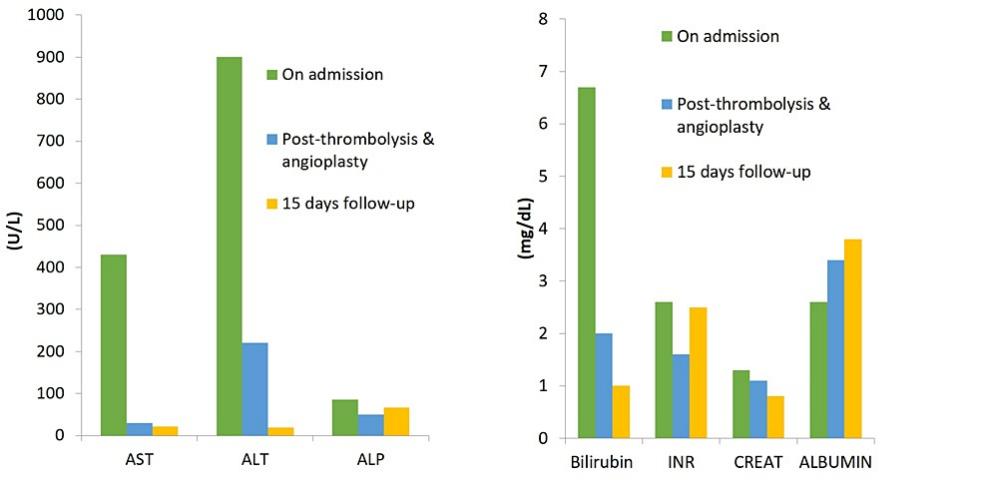
Bar chart showing normalization of liver enzymes, bilirubin, and albumin after thrombolysis. The patient was put on warfarin to maintain INR at 2.5. INR, international normalized ratio.

Powerflex 10 x 20 mm balloon and 10 x 60 mm balloon (Cordis, USA) were used for angioplasty followed by deployment of a Epic 10 x 60 mm self-expanding stent (Boston Scientific, USA). The stent was deployed so that a small portion of the stent extended across the ostium into the IVC. Subsequently, progressive improvement in liver function tests was documented. Two weeks after thrombolysis and angioplasty, the levels of liver enzymes, bilirubin, and albumin returned to baseline (Figure 5).

**Figure 6:**
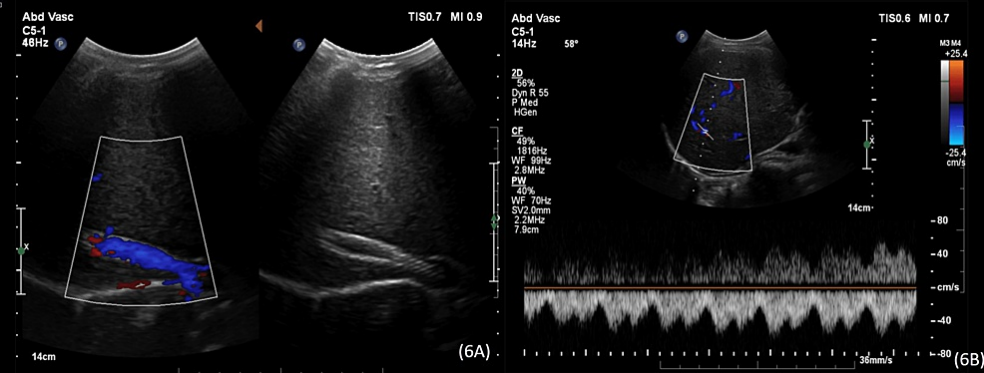
Five-year follow-up ultrasound and color Doppler images show patent right hepatic vein with normal color flow and stent in situ (6A) and normal periodic waveform pattern on spectral Doppler (6B). The liver architecture is normal.

Pro-coagulant work-up showed JAK-2 mutation; however, there was no evidence of any myeloproliferative disorder. The patient was discharged on a titrated dose of warfarin to maintain INR at 2.5. Serial follow-up ultrasounds at six-month interval demonstrated patent stent and normal venous flow. Doppler ultrasound performed at a five-year follow-up showed a patent right hepatic vein with the stent in situ and normal right hepatic venous waveforms (Figure 6A, 6B).

## Discussion

Therapeutic management of BCS depends on various factors such as duration (acute versus chronic), the severity of liver dysfunction, and anatomical site of obstruction (hepatic vein versus IVC). Thrombolytic therapy, angioplasty with or without stenting, transjugular intrahepatic portosystemic shunt (TIPS), direct intrahepatic portosystemic shunt (DIPS), portocaval shunting, and liver transplantation are the management options described in various studies [4-8]. Early thrombolytic therapy is crucial to the management of acute BCS. A review by Sharma et al. showed that early thrombolytic therapy is helpful in the recanalization of the hepatic veins if the thrombolytic agent is infused directly into the hepatic veins and is combined with angioplasty with or without stenting to achieve luminal patency. Successful thrombolytic therapy necessitates early administration of the thrombolytic agent, targeted catheter infusion, and combined angioplasty [5]. The study also showed that systemic administration of thrombolytic agents was of limited value. Thrombolytic therapy without angioplasty was also prone to re-thrombosis and poor outcomes. Thrombolytic therapy has the highest success rates in patients with acute BCS and partially occlusive thrombus of the hepatic veins [5]. Partially occlusive thrombus allows navigation of the thrombus with a catheter and maximum contact of the thrombolytic agent with the thrombus. Beckett et al. highlighted the role of hepatic venous angioplasty in the management of acute BCS in order to prevent long-term liver damage [6]. Another study by Zhang et al. has also shown good clinical outcomes in patients of acute BCS treated with catheter-directed thrombolysis and angioplasty [9].

The dosage of the different thrombolytic agents has been standardized in the management of arterial thrombosis including peripheral and systemic arteries [10,11]. However, there is no standardized protocol regarding the choice of a thrombolytic agent, and the dose and duration of local infusion in the setting of acute BCS. Previous studies have used different thrombolytic agents like streptokinase, urokinase, and alteplase with the duration of infusion ranging from 48 to 72 hours [12-15]. Zhang et al. have used urokinase for prolonged thrombolysis with an average duration of 5.2 ± 1.7 days [9]. In our case, alteplase was infused for a total duration of 84 hours into the thrombus. Based on the observations in our case, catheter-directed infusion of the thrombolytic agent in a slow flow system like the hepatic vein for more than 72 hours may lead to improved recanalization and reduced risk of re-thrombosis. Prolonged administration would be justified if adequate thrombolysis is not achieved within 72 hours, there is progressive interval improvement on intermittent check runs, and there are no bleeding complications. This is because adequate blood flow and low resistance are required for successful diffusion of the thrombolytic agent, which may not be possible within the limited-duration thrombolysis procedure in a slow flow system such as the hepatic venous system in the presence of acute thrombotic obstruction. Therefore, increasing the duration of infusion for successful recanalization of the hepatic veins will not only increase the success rate of subsequent angioplasty and stent placement but also avoid the need for TIPS/DIPS and liver transplantation.

We used an on-table modified thrombolysis catheter as demonstrated by Ram et al. in their study [16]. Successful recanalization of one of the three hepatic veins is sufficient for the clinical resolution of BCS [6]. Prior cross-sectional imaging should be performed to select the best hepatic vein for the recanalization procedure. In our case, we selected the right hepatic vein because of the predominant involvement of the right hepatic lobe and the relatively straight course of the right hepatic vein. Hepatic veins can be accessed via transjugular, transfemoral, or transhepatic approach. The transjugular route is the preferred route. A combined transhepatic and transjugular route is used when the hepatic venous ostium is not accessible from the IVC [6].

Thrombolytic therapy and angioplasty for hepatic venous obstruction is an effective and safe method for the management of acute BCS [5,9]. Although thrombolysis carries the risk of bleeding, the risk is higher in older patients while the majority of BCS patients are young. Hence, the procedure is not associated with serious bleeding complications when performed for BCS [5]. After successful recanalization, a significant and rapid improvement in symptoms and liver function has been observed [16]. In this procedure, there is no diversion of portal venous flow and, therefore, no risk of post-procedural encephalopathy. Restenosis is more commonly observed when angioplasty is performed in isolation as compared to angioplasty and stenting. Restenosis can be treated with balloon dilatation of the occluded segment and stenting.

TIPS is performed in chronic BCS when the patient is symptomatic and hepatic vein recanalization cannot be achieved or if there is a persistence of symptoms after thrombolysis and angioplasty. TIPS procedure is challenging to perform in cases where the main hepatic vein is replaced with intrahepatic collaterals or when the ostium of the hepatic vein cannot be identified. In such cases, DIPS or portocaval shunting (between the portal vein and IVC) is performed [4,6]. In cases of BCS, there is an increased risk of thrombosis and stenosis of TIPS; if this complication occurs, then thrombolysis and balloon dilatation of the stenosed segment can be performed [5].

Liver transplantation can be avoided in BCS in cases successfully managed with hepatic vein recanalization or TIPS procedure [6]. Radiologic interventions for BCS have been proven to improve liver function remarkably and a good midterm transplant-free survival [17]. Patients receiving anatomic recanalization show improved liver synthetic functions compared with patients treated with portosystemic shunts [17]. Liver transplant in BCS is reserved for patients with end-stage liver disease or fulminant hepatic failure, those who cannot be managed by recanalization or TIPS/DIPS procedure, or in cases where these procedures have failed [4,6]. Recurrence of BCS in the transplanted liver can also occur [4,6]. 

**Figure 7:**
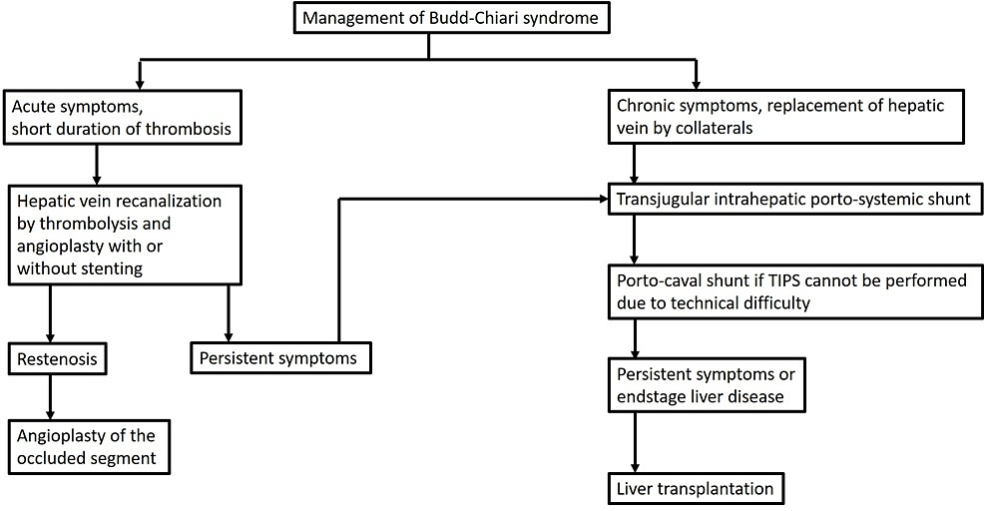
Flowchart demonstrating management protocol of Budd-Chiari syndrome. TIPS, transjugular intrahepatic portosystemic shunt.

It is extremely important to identify the causal pro-thrombotic event in cases of acute BCS. Anti-coagulation therapy should be initiated and continued indefinitely in patients with permanent risk factors for thrombosis [18]. A target INR of 2.5 should be achieved. This will improve long-term success rates and prevent thrombosis of the recanalized segment [4]. Oral contraceptives are contraindicated in patients of BCS. Long-term follow-up every three months in the first year and then annually using ultrasound and color Doppler should be done for early detection of re-thrombosis [4]. Our institutional management protocol for BCS is outlined in Figure 7.

## Conclusions

It is important to identify acute BCS in cases of acute hepatic failure and perform prompt recanalization of the thrombosed hepatic veins with prolonged catheter thrombolysis, angioplasty, and stenting. This may reduce the requirement for TIPS and liver transplantation in such cases and result in good long-term outcomes. However, large multicenter trials are required to further validate the results.
